# Efficient Ray Tracing of Large 3D Scenes for Mobile Distributed Computing Environments

**DOI:** 10.3390/s22020491

**Published:** 2022-01-10

**Authors:** Woong Seo, Sanghun Park, Insung Ihm

**Affiliations:** 1 Department of Computer Science and Engineering, Sogang University, Seoul 04107, Korea; wng0620@sogang.ac.kr; 2 Department of Multimedia, Dongguk University, Seoul 04620, Korea; mshpark@dongguk.edu

**Keywords:** mobile distributed computing, mobile GPU ray tracing, augmented and mixed reality, space-efficient 3D scene representation, master/worker system

## Abstract

Cluster computing has attracted much attention as an effective way of solving large-scale problems. However, only a few attempts have been made to explore mobile computing clusters that can be easily built using commodity smartphones and tablets. To investigate the possibility of mobile cluster-based rendering of large datasets, we developed a mobile GPU ray tracer that renders nontrivial 3D scenes with many millions of triangles at an interactive frame rate on a small-scale mobile cluster. To cope with the limited processing power and memory space, we first present an effective 3D scene representation scheme suitable for mobile GPU rendering. Then, to avoid performance impairment caused by the high latency and low bandwidth of mobile networks, we propose using a static load balancing strategy, which we found to be more appropriate for the vulnerable mobile clustering environment than a dynamic strategy. Our mobile distributed rendering system achieved a few frames per second when ray tracing 1024 × 1024 images, using only 16 low-end smartphones, for large 3D scenes, some with more than 10 million triangles. Through a conceptual demonstration, we also show that the presented rendering scheme can be effectively explored for augmenting real scene images, captured or perceived by augmented and mixed reality devices, with high quality ray-traced images.

## 1. Introduction

Since the emergence of mobile devices such as smartphones and tablets, the computing environment has been rapidly shifting from personal computers (PCs) to mobile platforms. However, despite the remarkable improvements in computing power, it remains a challenge to effectively solve a computationally intensive, large-scale problem on a single mobile device, particularly when a fast response is needed. Whereas the use of high performance compute servers is often suggested as a practical solution, only a little research has been conducted on the idea of exploring mobile computing clusters that consist of easily available mobile devices connected through mobile and wireless communication networks. In particular, achieving interactive distributed rendering on mobile clusters for generating high quality images from large three-dimensional (3D) scenes also remains a difficult problem to be tackled with great effort.

In spite of its high computational cost, ray tracing has been popularly used to produce significantly more realistic images than conventional rasterization methods [1]. In contrast to the rasterization-based renderers that adopt only approximate (often inaccurate) rendering models, ray tracing is capable of creating various advanced rendering effects, such as shadow, reflection, refraction and diffuse interreflection, in a physically correct manner. Note that such ray tracing effects could enhance immersive experiences markedly for augmented reality (AR) or mixed reality (MR) users who utilize mobile extended reality (XR) devices such as smartphones/tablets (for AR users) and a Microsoft HoloLens headset (for MR users). This is possible by augmenting the real scene image, which is either captured by the camera of an AR device or perceived via a see-through holographic lens of an MR device, using optically-correct realistic ray-traced images. (Blending the real scene images with carefully synthesized information is a key method to increasing spatial presence in AR and MR applications. Please refer to related work, e.g., [2,3,4].) These days, the dedicated ray tracing hardware of the graphics processing unit (GPU) often allows efficient acceleration of the ray tracing computation on the PC platform [5]. However, despite many efforts to develop an effective mobile ray tracing accelerator (refer to, e.g., [6]), ray tracing is still regarded as computationally burdensome on mobile platforms, mainly due to its excessive power consumption and heating problems.

This work was concerned with exploring the possibility of mobile cluster-based distributed computing as an effective mechanism for ray tracing large 3D scenes that consist of a few million to 12 million triangles. We were especially interested in investigating how an interactive mobile distributed ray tracing technique can be combined with mobile XR devices for improving the visual realism of the images that are acquired by them. More specifically, this paper presents a mobile distributed GPU ray tracing scheme that is well suited for rendering large 3D scenes with many millions of triangles, aiming at interactive speeds on a small-scale mobile cluster. Note that to the best of the authors’ knowledge, this was the first work to attempt to interactively ray trace large 3D scenes having more than ten million triangles on a cluster system made of commodity mobile devices.

The main contributions of this paper are as follows. First, to allow a mobile device to load as large a 3D scene as possible into GPU memory of limited size, we present a space-efficient 3D scene representation technique that is specially designed for mobile GPU ray tracing (Section 3). Note that sending bulky 3D scene data on the fly between mobile processors during interactive distributed rendering is inappropriate on the current mobile clusters because of the high transmission latency. Therefore, our mobile distributed ray tracing system employs a tile-based rendering scheme in which a mobile device stores a full copy of all needed 3D scene data encoded in space-efficient data structures. Then, each mobile device that participates in distributed ray tracing renders repeatedly assigned tile areas, returning the resulting tile images to the master device. To allow this, we present a 3D geometry encoding scheme that reduces the size of triangular meshes markedly at very little extra decoding cost for repeated random access on mobile devices with low processing power and memory bandwidth (Section 3.1). Combined with the kd-tree encoding scheme that was proposed by Seo et al. [7] (Section 3.2), we show that both the triangular mesh and the essential kd-tree structure of such a large Power Plant scene with more than 12 million triangles can be successfully loaded onto the GPU memory of a low-end mobile device for efficient distributed ray tracing.

Second, we evaluated two different distributed rendering mechanisms to find the best model for effective mobile cluster-based ray tracing in the current mobile and wireless network environment (Section 4). We found that the presented static load balancing scheme is more efficient than the dynamic scheme in the mobile clustering environment, which suffers from low bandwidth and high transmission latency. The performance of our mobile distributed ray tracer is described in detail in the results section (Section 5). In particular, we show that large 3D scenes with a few million to 12 million triangles can be rendered at a few frames per second to create ray tracing images of 1024×1024 pixels on a small-scale cluster made of 16 low-end smartphones. We also demonstrate via a proof-of-concept implementation how the presented mobile cluster-based ray tracing method can be utilized to enhance the visual realism of images that are displayed on the screens of wireless AR and MR devices. Then, the paper is concluded in the final section (Section 6).

## 2. Related Works

Thanks to the advances in mobile technology, mobile cluster computing has emerged as a prospective distributed computing paradigm that can effectively be used to solve large-scale problems [8]. Ray tracing of large-scale 3D scenes and/or scientific datasets is a good option for mobile cluster computing that requires a nontrivial amount of computational resources. Until several years ago, it was believed that mobile platforms could not be used to directly run applications with intensive rendering workloads. Therefore, remote rendering systems that rely on high performance servers were often explored (refer to previous work, e.g., [9,10,11,12,13]), where hiding or reducing the high communication latency caused by mobile and wireless networks between mobile clients and servers was an important issue. Collaborative rendering systems that utilize multiple mobile GPUs were also built, e.g., [14], but they were not appropriate for applying to mobile distributed ray tracing, which this paper intends to address.

Ray tracing demands a large amount of computation, particularly for building spatial acceleration structures and calculating ray–object intersections. Therefore, there have long been attempts to develop dedicated hardware that accelerates the essential ray tracing computations. Modern high-end PC GPUs often enable real-time ray tracing for nontrivial 3D scenes [5]. On the other hand, despite the endeavors dedicated to developing a mobile accelerator for ray tracing [6,15,16,17], a single modern mobile GPU still does not provide enough performance for real-time rendering of large 3D scenes.

Distributed ray tracing can be an effective way of solving this problem. Previous research on interactive distributed ray tracing usually considered PC and/or workstation clusters (refer to, e.g., [18]). By contrast, very little work has been done to utilize a computing cluster built using ubiquitous mobile devices. Choosing a proficient load balancing strategy for parallel or distributed ray tracing is important, and dynamic load balancing has often been preferred over static one [19]. Recently, the load balancing issue was addressed in the mobile cloud computing environment [20]. A tile-based, distributed ray tracing system was developed on a small-scale mobile cluster, where only six devices were able to participate in the distributed rendering [7]. Unlike their approach, the method presented in this paper uses a space-efficient 3D scene representation combining both mobile GPU-friendly geometry and kd-tree encoding methods, which, as a result, allows it to load larger 3D scenes into GPU memory for effective mobile distributed rendering. Furthermore, our work employs a static load balancing strategy which turned out to be more efficient for mobile distributed ray tracing. Thanks to this policy, we can expand the number of participating worker devices up to 16 without significantly harming the distributed rendering efficiency.

## 3. Space-Efficient Representation of 3D Geometry for Mobile Distributed Ray Tracing

In our mobile ray tracing, we adopt the indexed-face-set format to describe the geometry of a given scene. Here, the corresponding triangular mesh is represented in two arrays that list vertex attributes and triangle vertex indices, respectively. The original scene data that we have ray traced used eight 32-bit floats per vertex for vertex attributes (three for position, three for normal and two for texture coordinate), and three 32-bit unsigned integers per triangle for triangle vertex indices, demanding $32n_{v}+12n_{f}\;\left(=8\times 4\times n_{v}+3\times 4\times n_{f}\right)$ bytes to represent a 3D geometry model with $n_{v}$ vertices and $n_{f}$ faces. In addition to the triangular mesh, we also employ a kd-tree, which is an essential spatial acceleration structure for real-time ray tracing. Coupled with the mesh data, this extra data structure also must be loaded into memory, whose size is usually in the order of that of the mesh data. For instance, for the Power Plant dataset with 10,960,555 vertices and 12,738,500 faces, the necessary memory space amounts to 480.6 MB (for triangular mesh) plus 603.7 MB (for kd-tree), which is quite burdensome to handle in mobile ray tracing (refer to Table 1). Therefore, we must find an effective way of representing 3D geometry, enabling efficient mobile distributed computing.

### 3.1. Reducing the Size of the Triangular Mesh

The mobile device is generally not able to hold the extensive 3D scene data in its memory space for real-time ray tracing. Therefore, it requires the usage of a space-efficient 3D geometry representation scheme. For the real-time rendering, particularly on a mobile device, a prospective space-reduction method should keep the extra cost of data fetching incurred by its data encoding scheme very low. There exist several 3D geometry compression methods in the literature that achieve substantial space reduction while offering rapid random accessibility [23]. For our application, however, we put more weight on minimizing the geometry decoding cost rather than raising the degree of space reduction. That is because the mobile device, on which the ray tracing computation is already not fast enough, is more vulnerable to a complicated decoding calculation than a PC due to the low processing power and memory bandwidth. In particular, after an experiment with existing compression techniques on the target smartphone, we found it more appropriate to quantize only the vertex attributes listed in the indexed-face-set representation. This decreases the space requirement markedly at very little extra decoding cost for repetitive random accesses.

#### 3.1.1. Quantization of Vertex Attributes

**Figure 1:**
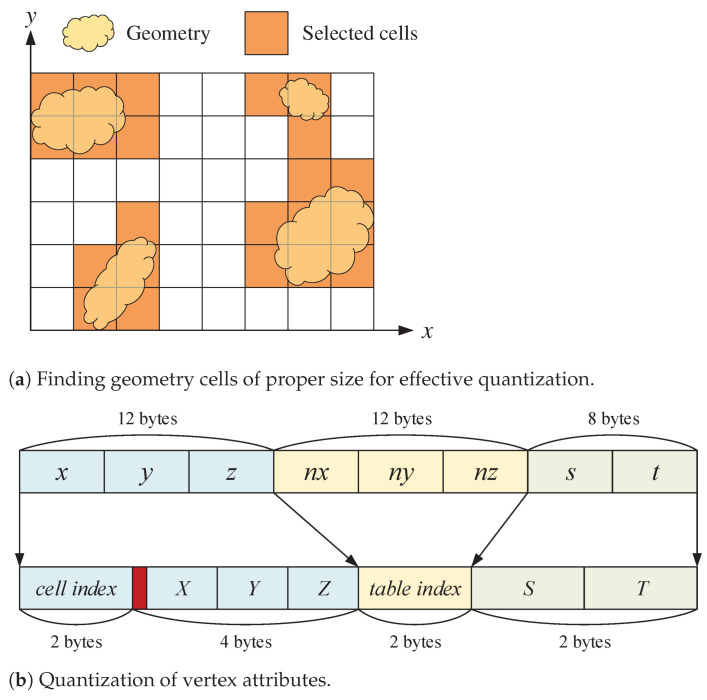
Quantization of 3D geometry data.

Construction of a cell index table. The first step toward our space-efficient mesh representation is to partition the (slightly enlarged) axis-aligned bounding box (AABB) of a given scene into equal-sized cubic cells, and find those cells, called the *geometry cells*, that are intersected by the scene’s triangular mesh (see Figure 1a). A key factor in partitioning the scene space is to set the size of the cubic cell to be as small as possible while the resulting number of geometry cells cannot exceed 65,536 (=$2^{16}$). This can be done by subdividing the longest side of the AABB repeatedly, in which the desired number of subintervals is decided in a bisection manner from an initial range, say, $\left[2^{5},2^{10}\right]$. After a proper cell size is determined, the geometry cells are then enumerated, storing their indices (i,j,k) in increasing order in a table, called the *cell index table*. Since the size of table is at most 65,536, a geometry cell can be indexed using a 16-bit unsigned integer.

Quantization of the position attribute. Once the scene space is tessellated as finely as possible using cubic cells, each side of the geometric cell is quantized into 1024 (=$2^{10}$) levels, at which the location of a vertex position within a cell is described. Since the exact location of the cell in the AABB is known through its index, this quantization allows us to represent a vertex position (x,y,z) using a cell index of two bytes plus three offsets (X,Y,Z) of 10 bits each, reducing the memory requirement from 12 bytes to 6 bytes (see Figure 1b). Note that the resolution of this space quantization method, i.e., the quantization step size, becomes $\frac{l_{c}}{1024}$, where the cell size $l_{c}$ is the length of the longest side of the scene AABB divided by the number of the corresponding interval partition (refer to Table 2).

Quantization of the normal attribute. Contrary to the vertex position each component of which is quantized within a cell, the vertex normal is quantized as a whole. For run-time access during rendering, we adopt a simple table-based quantization technique where a preprocessed lookup table, indexed with a 16-bit unsigned integer, provides up to 65,536 =$(2^{16})$ discrete unit normal samples. To fill the table, we use the normal encoding/decoding method of Smith et al. [24], which exhibits the lowest mean and maximum angular errors among a variety of 16-bit unit normal representation techniques [25]. Once a lookup table is prepared, the original 12-byte unit normal $\left(n_{x},n_{y},n_{z}\right)$ of a vertex is transformed into a two-byte index whose corresponding direction is closest to that of the original vector.

Quantization of the texture-coordinate attribute. The texture coordinates have often been compressed successfully using the predictive schemes developed for the vertex position, with a few variations (e.g., [26,27,28]). Unfortunately, however, such rather complicated methods are not well suited for mobile computing platforms. In fact, when the coordinates are normalized between 0 to 1, we find that a 16-bit unsigned integer that can quantize each component uniformly into 65,536 levels is sufficient for mobile ray tracing because most texture images have resolution smaller than 2048×2048 pixels. Note that for some scenes such as San Miguel, however, the texture coordinates may be in any range, permitting textures to be repeated. Even in such cases, however, the three texture coordinates from a triangle can usually be transformed safely into the range [0,2]×[0,2]. Therefore, we quantize each component of a texture coordinate (s,t) into two bytes of memory: the most significant bit stores the integer part, and the remaining 15 bits store the fractional part in 32,768 (=$2^{15}$) levels.

#### 3.1.2. Analysis of Triangular-Mesh Compression

Size reduction ratio. Note that for a triangular mesh in the indexed-face-set representation with $n_{v}$ vertices and $n_{f}$ faces, the original mesh dataset actually needs $32n_{v}+12n_{f}+8$ bytes of memory, among which the 8 additional bytes are for storing the values of $n_{v}$ and $n_{f}$ (a four-byte unsigned int each). On the other hand, the presented quantization method requires $12n_{v}+12n_{f}$ bytes (for the lists of quantized vertex attributes and triangle vertex indices) plus $3\cdot 2\cdot 2^{16}$ bytes (for the cell index table in which each index component is saved as a two-byte short integer), $3\cdot 4\cdot 2^{16}$ bytes (for the normal lookup table in which each normal component is saved as a four-byte float) and 24 bytes (for the values of $n_{v}\left(4\right)$, $n_{f}\left(4\right)$, the xyz coordinates of AABB’s anchor point(12), and the cell size(4)). This leads to the size reduction ratio of $\frac{32n_{v}+12n_{f}+8}{12n_{v}+12n_{f}+18\cdot 2^{16}+24}$ = $\frac{8n_{v}+3n_{f}+2}{3(n_{v}+n_{f})+294918}\approx 3+\frac{5}{1+n_{f}/n_{v}}÷3+\frac{294918}{n_{v}+n_{f}}$, which will generally increase when the size of mesh—i.e., $n_{v}+n_{f}$ increases. Furthermore, the ratio gets higher as the ratio between $n_{v}$ and $n_{f}$, i.e., $\frac{n_{f}}{n_{v}}$, decreases. As reported in Table 3, the quantization method achieves the ratios of 1.69 (Sponza) and 2.10 (Soda Hall) for the four test scenes, for which the ratios are more affected by the parameter $\frac{n_{f}}{n_{v}}$ (refer to Table 1 again for the numbers of vertices and faces).

Visual artifacts in ray tracing. Whereas the presented quantization method effectively improves the space efficiency, it inevitably decreases the image quality due to the quantization errors caused when the size-reduced triangular mesh is ray traced. To reveal how the geometry quantization negatively affects the rendering quality on the mobile device, we compare the ray-traced images of 1024×1024 pixels against the corresponding ground truth images obtained by ray tracing the original geometry. As shown in Table 4, when only the positional attribute is quantized (“POS” in the table), the resulting PSNR values are quite high, and it is very difficult to visually distinguish between those images.

**Figure 2:**
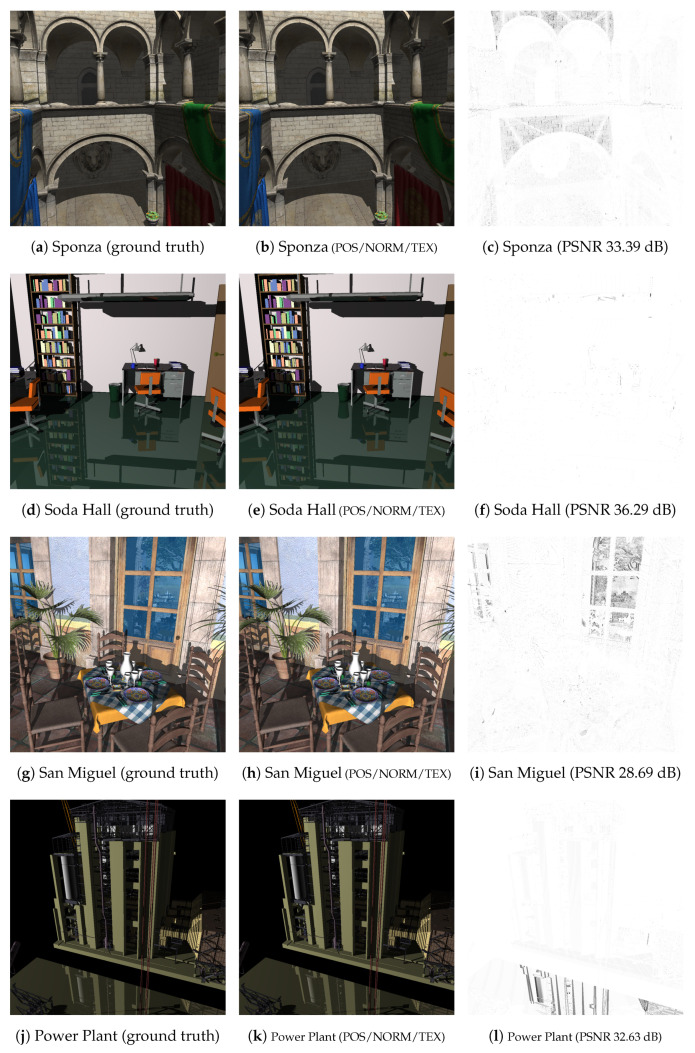
Comparison with ground truths. The left and middle columns display the images rendered by ray tracing the original and quantized triangular meshes, respectively. The right column then shows their differences with achieved PSNR values.

When the normal attribute is additionally quantized (“POS/NORM”), the PSNR values start to decrease. However, the degree of reduction varies among the tested scenes. For the Sponza and Soda Hall datasets, whose scene complexity is relatively low (279,163 and 2,167,474 triangles, respectively), this additional quantization does not accumulate noticeable visual artifacts in the ray-traced images (from 42.80 dB to 42.59 dB for Sponza and from 38.57 dB to 36.20 dB for Soda Hall). Note the the normal vector is the key element needed for local shading. More importantly for ray tracing, it is used to calculate the directions of secondary, reflection and refraction rays. When such scenes with high geometric complexity as San Miguel and Power Plant (9,963,191 and 12,748,510 triangles, respectively) are rendered, the small quantization error in the normal direction, and thus in the direction of secondary rays results in marked decreases in the PSNR value (from 34.94 dB to 29.17 dB for San Miguel and from 43.23 dB to 32.63 dB for Power Plant). This is because, for complex scenes with highly detailed geometry, the reflection/refraction rays, whose directions are slightly perturbed, often hit the surface points whose geometry is somewhat different from that for the correct secondary rays. This phenomenon is clearly shown in the difference images in Figure 2i,l, where the visual artifacts exist mostly on the reflective surfaces. (In the San Miguel scene, we set the bluish window glasses to reflective.) On the other hand, for the Soda Hall scene with relatively simpler geometry, the slightly changed secondary rays do not produce significant aliases in the ray-traced image (see the floor surface in Figure 2f). The effect of the slightly perturbed reflection/refraction rays can clearly be ascertained when those rays are not traced (“- no refl/refr” in Table 4). With these rendering features turned off, the resulting image quality is almost the same even though the normal attribute is quantized (from 35.17 dB to 35.13 dB for San Miguel and from 43.23 dB to 41.10 dB for Power Plant).

Finally, the negative effect of the additional texture-coordinate quantization on the image quality appears differently depending on the scene complexity and the texture properties (“POS/NORM/TEX”). If the scene geometry is rather simple but the applied textures are quite detailed, the additional quantization causes a considerable decrease in the PSNR value (e.g., from 42.59 dB to 33.39 dB for Sponza). On the other hand, if the scene geometry is complex but the textures are relatively less complicated, such as San Miguel, only a small decrease will be observed (from 29.17 dB to 28.69 dB). This means that the rendering artifacts are mainly due to the quantization errors from the position and normal attributes.

**Figure 3:**
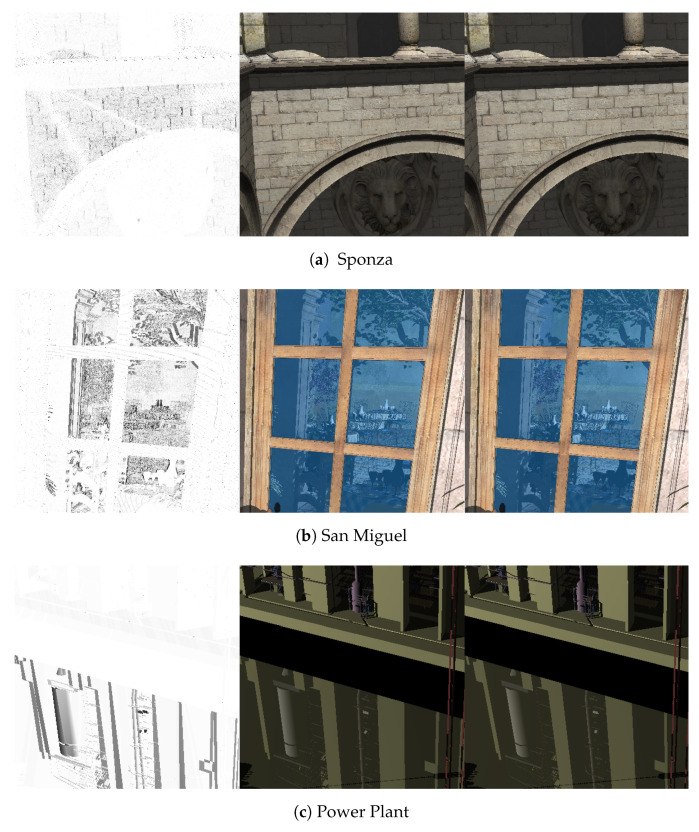
Comparison with ground truth images (partially enlarged). For each triplet of images, the ground truth image (**middle**) is compared with our result (**right**), obtained by ray tracing the original and quantized triangular meshes, respectively. Even in the most problematic areas, the visual difference is quite negligible.

Overall, we achieved PSNRs of 28.69–36.29 dB for the four example scenes when the fully quantized triangular meshes were ray traced. On the tested mobile devices, the visual difference between the rendering results and the ground truths was usually negligible at best both spatially and temporally. As compared in Figure 3, even in the most troublesome areas, the actual difference is hardly discernible.

### 3.2. Reducing the Size of the Spatial Acceleration Structure

#### 3.2.1. Enhancement of Memory-Space Efficiency of the kd-Tree

As mentioned before, the kd-tree is an efficient spatial data structure that provides fast ray–object intersection during ray tracing. However, when it is constructed from a triangular mesh using a standard recursive algorithm, a great number of triangles that intersect with splitting planes are repeatedly duplicated into a large number of leaf nodes. This frequently results in inefficient, large and tall trees with high triangle redundancy, which are burdensome to handle, particularly on mobile platforms (see Table 1 again). To improve the memory-space efficiency of kd-trees, Choi et al. [29] extended the de facto standard kd-tree construction and representation algorithms [21,22] in such a way that an inner node of the tree may optionally keep a pointer to a triangle that would otherwise be repeated in an excessive number of leaf nodes. Coupled with a modified kd-tree traversal algorithm, the new kd-tree scheme was shown to significantly decrease the memory requirements for representing the kd-tree structure with only a slight increase in the tree traversal cost during ray tracing.

Reducing the kd-tree size further was necessary for mobile devices with limited GPU memory space. Therefore, Seo et al. [7] proposed to allow an inner node to store up to two triangle references when excessive triangle duplication is found. This simple modification is possible because in the extension by Choi et al., each inner node is stored with 8 byte alignment for efficient caching, and therefore the lower 4 bytes of the inner node that refer to a triangle are not used. In fact, allowing an additional triangle reference in an inner node may cause more frequent calculations of early ray–triangle intersection during the kd-tree traversal, although many of them are actually unnecessary. Since they naturally increase divergent branches during ray tracing, the strategy of allowing up to two triangles per inner node may slow down the ray tracing computation significantly on a PC GPU. Therefore, in Choi et al.’s method targeting high performance PC GPUs, at most one triangle reference is allowed per inner node. In contrast, mobile platforms suffer from limited graphics memory and memory bandwidth, in addition to insufficient parallel processing capabilities. Therefore, the advantage of saving memory space by storing two triangle references outweighs the disadvantage of slowing down the rendering computation, as will be shown shortly.

#### 3.2.2. Analysis of kd-Tree Compression

In this subsection, we briefly describe the performances of the three kd-tree construction methods: standard [21,22], Choi et al. [29] and Seo et al. [7]. For a fair comparison of the two modification algorithms by Choi et al. and Seo et al., we applied the same occupancy and frequency parameters $\left(\tau_{occu},\tau_{freq}\right)=\left(0.5,0.4\right)$ during the kd-tree construction for the four nontrivial example scenes. (Please refer to [29] to understand how these parameters control the properties of generated kd-trees.) In addition, we allowed up to eight triangle references in total along the inner nodes on the path from every leaf node to the root node, which compromises well between the tree size reduction and ray tracing speed. Table 5a first compares the performances in view of size reduction of the kd-trees generated. Seo et al.’s method, which is included in our system, exhibited a compression ratio of 1.98 to 3.03 with respect to the standard method. Considering the compression ratio of Choi et al.’s method, which ranged between 1.58 and 2.18, we notw that the idea of allowing an inner node to point to another redundant triangle is quite effective at reducing the size of kd-trees further. When Seo et al.’s method is applied, the size of the indexing data also decreases slightly, as removing redundant triangles from leaves improves spatial coherence in the triangle index lists.

Note that, among the four test scenes, Seo et al.’s method had the greatest size reduction for the San Miguel scene in comparison to both the standard method and Choi et al.’s method. The San Miguel dataset contained a significant number of ill (possibly long and skinny) triangles that were intersected repeatedly with the splitting planes during kd-tree construction. Therefore, moving a next most redundant triangle from leaf nodes to an inner node still resulted in a substantial amount of size reduction. Refer to Table 5b to see how effectively the number of kd-tree nodes decreases when the new extension is applied in addition to Choi et al.’s method.

As noted before, an additional triangle reference at an inner node inevitably causes the ray tracing frame rates to drop to some extent because of the extra computation for early, possibly unnecessary, ray–triangle intersection. However, the timing results in Table 5c reveal only a slight decrease in rendering speed compared to Choi et al.’s method, which is quite encouraging considering the increased complexity of the kd-tree traversal algorithm.

## 4. Distributed Ray Tracing Framework for Mobile Cluster Computing

The proposed mobile distributed ray tracing system is based on a master/worker model, in which a master device (client) controls multiple wirelessly-connected worker devices (servers) that perform actual distributed rendering. An important key in this system is to make each worker node hold all necessary scene data, including the triangular mesh and the kd-tree structure, in its graphic memory to minimize data transmission during distributed rendering. This is possible thanks to the presented space-efficient representation of 3D geometry and kd-tree structure. As is frequently done, the entire image space is partitioned into a grid of two-dimensional (2D) tiles, which becomes a pool of rendering tasks. In each time frame, the master first sends 6-degree-of-freedom camera poses and then tile indices on the fly to respective workers. It also collects rendered tile images from the workers to build the final ray-traced image. All rendering calculations are carried out on the GPUs of the workers against the tile indices assigned by the master.

Usually, remote rendering assumes a high-performance server system whose computing power well outperforms that of the client device. In contrast, the distributed rendering system in this work comprised a set of worker devices whose computing power was as limited compared to that of the master device. Therefore, it is important to carefully design an appropriate architectural and interaction model for efficient mobile distributed rendering. This will effectively cope with such constraints as the limited processor power and memory space of mobile devices, and the high latency and low bandwidth on networks in mobile environments. In this section, we describe two different models and show how they performed in the experiments.

### 4.1. Strategy I: Dynamic Load Balancing

Our distributed rendering system may be designed in such a way that only one thread on each side of the master and worker devices processes all tasks, such as communication and rendering. However in the mobile environment, the communication latency between wirelessly connected devices is significantly high and often hard to predict, causing a major bottleneck in the resulting distributed rendering system. Note that with a single thread on each side, the GPU ray tracing must halt while the rendered tile image is sent to the master. This leads to a substantial inefficiency in the usage of computational resources, suggesting that hiding the latency of mobile communication must be one of the most important factors in designing an effective mobile distributed rendering system.

**Figure 4:**
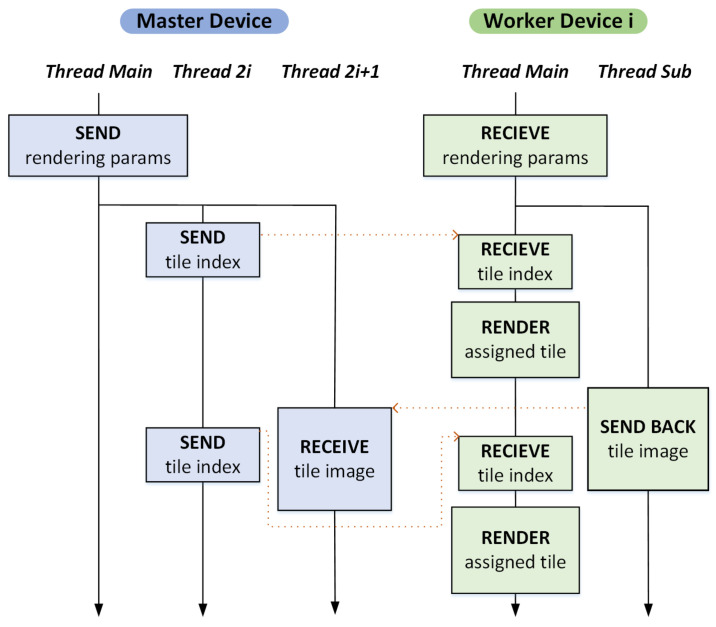
Master–worker interaction model I ([7]). This framework, based on dynamic load balancing, significantly improves the computational efficiency of mobile distributed rendering by separating the tasks of image transmission and GPU ray tracing in the master and worker nodes.

Figure 4 illustrates the interaction model that was previously proposed by Seo et al. [7]. Here, in order to overlap the tasks of data transmission and rendering, two extra threads per worker device are run on the master device along with the main thread (Thread Main). The first one (Thread 2i) is aimed at sending a tile index when the *i*th worker node is ready to render the next tile, whereas the second one (Thread 2i+1) is responsible for collecting the ray-traced tile images that the *i*th worker device sends. On the other hand, the corresponding *i*th worker node uses an extra thread (Thread Sub) so that the rendered tile image is transmitted, while the main thread (Thread Main) renders the next tile image on the GPU simultaneously. By performing the GPU ray tracing and the data communication in parallel as much as possible, the frame rate of the mobile distributed rendering increases markedly, as shown by Seo et al. [7]. In this distributed rendering scheme, the image tiles are distributed among the worker processors dynamically and adaptively according to their rendering capability, which may vary device by device.

### 4.2. Strategy II: Static Load Balancing

**Figure 5:**
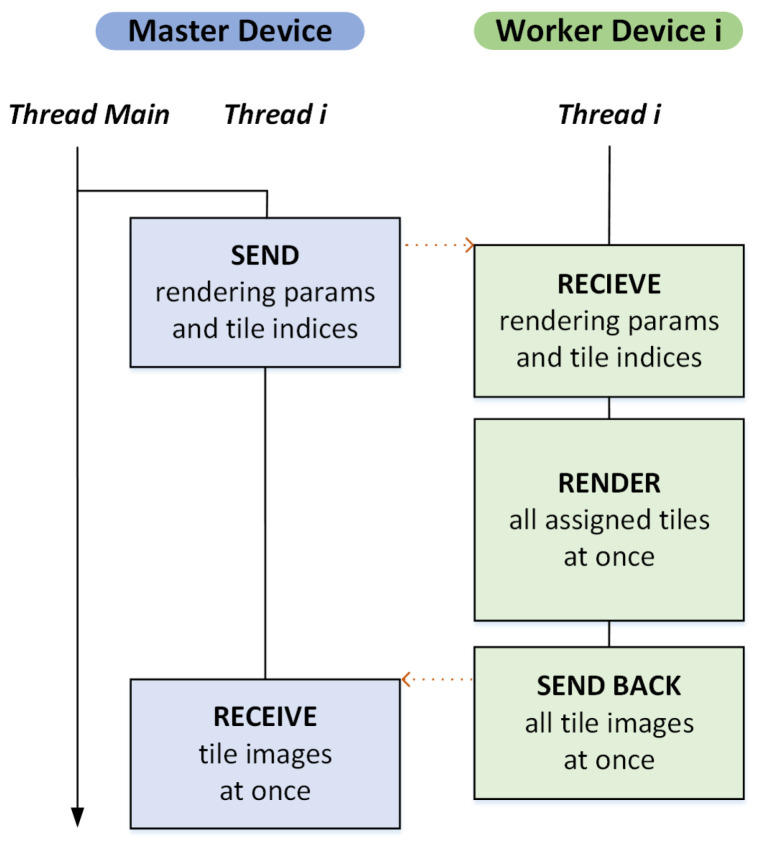
Master–worker interaction model II. As more workers participate, the increased communication between mobile devices becomes the dominating factor that harms the efficiency of distributed rendering. To overcome the problem, we propose adopting a simpler approach using static load balancing. Interestingly, the uneven load distribution across workers paradoxically improves the efficiency by effectively overlapping the tasks of image transmission and GPU ray tracing.

The distributed rendering model in the previous subsection aims to offer a flexible way of dynamic load balancing, which is effective for a small group of workers. However, the high latency and low bandwidth nature of mobile and wireless network connection often decrease its efficiency rapidly as more worker devices participate in the rendering. To overcome the problem, it is quite important to reduce the frequency of data communications between the master and workers as much as possible to avoid the transmission efficiency from deteriorating due to the high latency. Our preliminary experiment exhibits that, for the worker side, keeping rendered tile images and sending them together at once to the master is markedly more efficient on a mobile or wireless network than sending each image separately right after it is rendered. Therefore, in the second distributed rendering model, whose master–worker interaction model is illustrated in Figure 5, we have the workers wait until all assigned tiles are rendered, transferring the collected tile images once.

**Figure 6:**
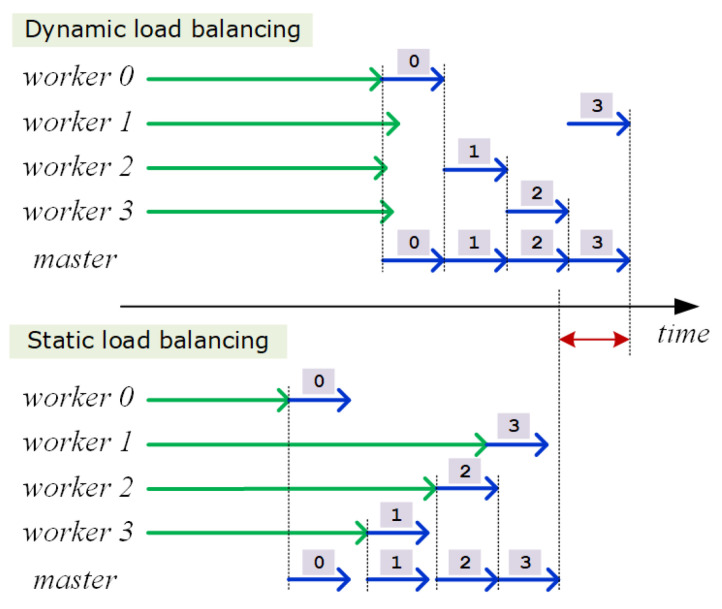
The situation in which static load balancing is faster than dynamic load balancing. For mobile wireless networks where the communication overhead is quite high compared to the workload, the static load balancing method turns out to be often faster in distributed rendering.

A problem with this approach is that the low bandwidth of the master device greatly reduces the efficiency in data transmission when multiple workers attempt to send the rendered images concurrently. For the tested mobile phone, the data transmission from multiple workers tended to get serialized, incurring a significant overhead during tile image collection. Paradoxically, balancing the rendering workload evenly often limits the efficiency of the distributed computing system because most of worker nodes try to transmit the tile images back to the master simultaneously. Interestingly, we found that disturbing the workload balance between the workers often increases the overall frame rate of the distributed rendering. This is because the rendering computation and the data transmission tend to get overlapped at least partially, amortizing the data transmission cost over the distributed rendering computation (refer to Figure 6).

Note that, particularly for mobile distributed rendering, the inefficient data transfer acts as a critical factor that degrades system performance more than rendering calculations do. In fact, we observed that even the trivial task of the master that distributes tile indices dynamically to the respective workers causes unignorable degradation in performance due to the high latency in data transfer. Hence, to maximize the throughput of the rendering system, the second distributed rendering model adopts a static load balancing strategy in which each worker sends back the tile images together once the whole rendering assignment is taken care of. In this scheme, however, to avoid overloading a few workers excessively, the entire image is subdivided into a collection of small tiles of given size, and assigned statically to the respective workers in round-robin manner.

## 5. Results

To demonstrate the effectiveness of the presented mobile distributed ray tracing scheme, we first implemented a full ray tracer that enables one to support the respective size-reduction methods for the triangular mesh and kd-tree. The ray tracer was then optimized on the mobile GPU with the OpenCL 2.0 API, in which work-groups of 8×8 work-items were applied for measuring timings. The tested mobile cluster of worker devices (servers) was made of a LG G5 smartphones, each of which used the Qualcomm Snapdragon 820 chipset equipped with the Adreno 530 GPU and was connected to an IEEE 802.11ac-based wireless network. On the other hand, an LG V50 smartphone adopting the Qualcomm Snapdragon 855 chipset and the Adreno 640 GPU was used as the master device (client).

**Figure 7:**
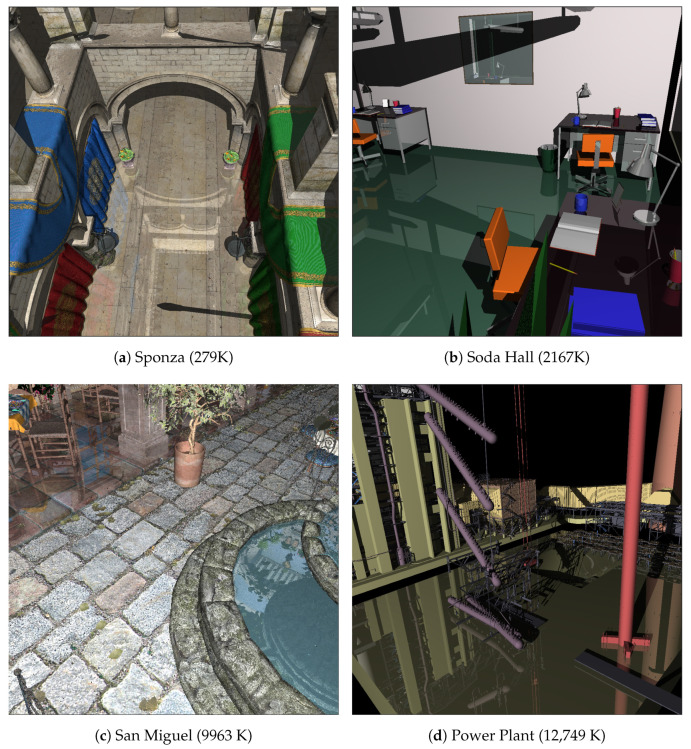
Example scenes and camera views tested. The figures in parentheses indicate the numbers of triangles in the respective scenes.

For the worker device, a graphics memory allocation error is encountered when trying to load rendering data larger than roughly 950 MB. Therefore, it would not have been possible to ray trace the San Miguel and Power Plant scenes without applying the presented size reduction techniques. In particular, San Miguel required the extra 226 MB of memory space for loading its texture images. Therefore, in the experiment, this scene was rendered without textures when needed. For consistent evaluation, all rendering times were measured at a resolution of 1024×1024 pixels with respect to the camera views in Figure 7.

### 5.1. Spatial and Temporal Performances of Size Reduction Methods

Table 6 summarizes how much the size of the original triangular mesh and kd-tree was reduced for effective mobile ray tracing. As explained before, the vertex attributes of the triangular mesh in the indexed-face-set format were compressed uniformly, storing each of 32 bytes in 12 bytes. In contrast, since the face information, i.e., the list of the vertex indices of triangles, was not compressed to minimize the decoding cost for random accesses, the degree of triangle mesh compression depended on the scene data (refer again to Table 3). On the other hand, the size reduction effect for the kd-tree was greater when, as in San Miguel (see Table 5a,b), the scene contained more triangles that frequently intersect with the axis-aligned splitting planes selected during the kd-tree construction. Combined together, our size reduction methods achieved compression ratios of over 2:1 for the three nontrivial scenes, permitting us to load the San Miguel and Power Plant scenes into the GPU memory of the tested smartphone for effective distributed rendering.

As emphasized before, rather than maximizing the compression ratio, we focused more on keeping the extra cost for accessing the compressed triangular mesh and kd-tree from increasing significantly after compression. This is particularly critical for efficient ray tracing on a mobile device with limited processing power and memory bandwidth. Table 7 reveals the additional cost incurred by the geometry compression during distributed ray tracing. When one worker device participates in rendering, the combination of the two size reduction methods leads to up to a 15.7% of increase in computation time compared to the uncompressed case (Table 7a).

Interestingly, this decreased significantly from 10.4% and 15.7% to 6.3% and 8.2% for Sponza and Soda Hall, respectively, when 16 worker devices take part in distributed rendering (Table 7b). Although we can not render the San Miguel and Power Plant scenes without compression, such a trend is also found clearly from the measured timings for these large scenes. This improvement in efficiency is presumed to have been because the extra cost of handling the compressed geometry was amortized with other computations of the distributed rendering. To wrap up, we confirm that the presented size reduction methods impose only a small burden (less than 10%) on the mobile distributed ray tracing.

### 5.2. Performances of Mobile Distributed Ray Tracing

**Figure 8:**
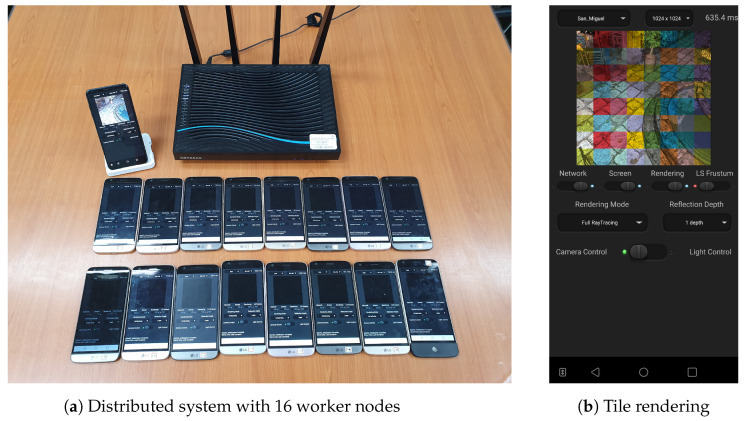
Our mobile distributed ray tracing system in action. (**a**) Sixteen worker devices participated in the distributed rendering of the San Miguel scene with high complexity. (**b**) Tile images that were produced by different worker nodes are overlaid in different colors to show the work distribution.

The size of the tile in the tile-based distributed ray tracing affects the rendering time significantly, particularly in a mobile environment. In an effort to find an optimal size, we measured the ray tracing times with varying sizes of tiles (see Figure 8). As shown in Table 8, the tile of 128×128 pixels usually produced the best performance, although not always: when the tile was subdivided further into 64×64 and 32×32 pixels, the distributed rendering time tended to increase rapidly. When more smaller tiles were assigned to a worker node, the ray tracing kernel needed to be launched on the GPU more frequently; relatively higher overhead of launching kernels on the mobile GPU suggests not using tiles of a size smaller than 128×128. Furthermore, in our tile-based distributed rendering, the tiles are enumerated row by row and then distributed to the workers in round-robin manner. Therefore, the locality of reference to scene geometry during ray tracing becomes worse for smaller sizes of tiles, which also negatively affects the distributed rendering performance.

Finally, Table 9 compares the two load balancing schemes that are proposed in this work. This experimental result where five runs were repeated to get each average rendering time reveals how efficiently the distributed rendering time reduces while increasing the number of participating worker machines. Here, the numbers in parentheses show the efficiency that is defined as $\frac{\tau_{1}}{S\tau_{S}}$, in which $\tau_{1}$ and $\tau_{S}$ represent the execution times with one worker and with *S* workers, respectively.

As shown in the tables, the achieved efficiencies were observed to be similar between the two load balancing schemes until up to four worker nodes were used. However, as eight nodes participated in the distributed rendering, the static strategy started to outperform the dynamic one. When the number was doubled to 16, the difference in the efficiency turned out to be significant among the two strategies. Particularly for the nontrivial scenes other than Sponza, the efficiency value from the dynamic load balancing strategy is less than a half of that from the static one. This is because the communication overheads grow very fast as image tiles of smaller sizes are transmitted more frequently on the fly through a mobile network. On the other hand, the static load balancing scheme was able to achieve better efficiency by amortizing the data communication cost over the distributed rendering computation, as explained before. Specifically, the static model was able to get efficiencies of 57.9% to 61.3% for the large scenes with 2167 K to 12,749 K triangles when 16 commodity smartphones participated in the distributed rendering. Considering the low-performance of the consumer-grade mobile devices with limited memory space and bandwidth and the high communication latency found in the mobile network environment, we believe the the presented ray tracing scheme provides an effective computational model for mobile distributed computing.

### 5.3. Augmenting Images Produced by Mobile AR and MR Sensors Using Ray Tracing

Handheld devices such as a smartphone and a tablet PC, and head-mounted displays such as Microsoft HoloLens, are routinely used to capture or perceive real scene images in augmented-reality (AR) and mixed-reality (MR) environments. The 6-degree-of-freedom (6-DOF) pose in real 3D space can be effectively tracked for the handheld devices using installed sensors such as an inertial measurement unit and/or a software toolkit such as Apple ARKit and Vuforia. In addition, the camera pose of the HoloLens headset can also be estimated faithfully in the 3D space using its built-in tracking system. In the previous experiment, the camera pose was selected using fingers on the touch screen of the smartphone. Addition, the capability of real-time tracking of camera poses enables one to combine these AR and MR devices with the presented mobile distributed ray tracing system to render high-quality ray-traced images on their displays.

**Figure 9:**
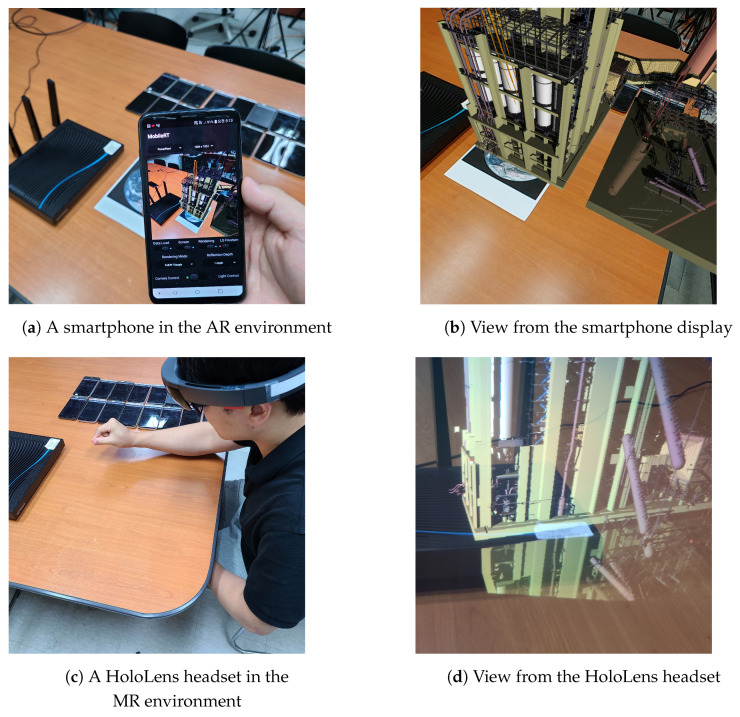
Mobile distributed ray tracingin AR and MR environments. Using the proposed mobile rendering system, it is possible to display ray-traced images at interactive frame rates on the displays of the tested AR and MR devices. By creating the optically-correct rendering effects such as shadow, reflection and refraction on these AR and MR devices, we can enhance the real scene images either captured by the AR device or perceived by the MR device, effectively augmenting the reality with 3D graphics.

Figure 9 shows our proof-of-concept implementation in which an LG G5 smartphone and a HoloLens headset functioned as master devices in the AR and MR environments, respectively, whose tracked camera poses were continuously sent wirelessly to the mobile cluster of worker devices. Then, the ray-traced images were sent back to the AR/MR devices for the display on their respective screens. Although the resulting frame rates were not sufficiently high for real-time rendering in the current test environments, we were able to successfully augment the real scene images via the advanced rendering scheme at interactive frame rates in the mobile AR and MR environments. Through the conceptual demonstration, we showed the possibility of mobile ray tracing where the ubiquitous mobile devices can help enhance the real scene images, either captured by the AR sensor or perceived by the MR sensor, using high-quality rendering images.

## 6. Concluding Remarks

In this paper, we have described a distributed ray tracing framework that is well suited to a mobile compute cluster made of consumer-grade mobile devices. The experimental results indicate that we can achieve a few frames per second on a small-scale mobile cluster in which 16 low-end smartphones participate when creating high quality images of 1024×1024 pixels against large 3D scenes having a few million to 12 million triangles. This is possible through the presented space-efficient scene representation scheme. For instance, both the triangular mesh and kd-tree of such a large 3D scene as Power Plant having more than 12 million triangles can be loaded onto 1 GB of GPU memory of a low-end smartphone. We have also shown how our mobile ray tracing scheme can be effectively coupled with XR applications where the images captured or perceived by AR and MR devices are nicely augmented with realistic ray tracing effects.

Currently, the frame rates achieved by the presented method are not high enough for real-time mobile ray tracing, which requires more than 30 frames per second. The most serious bottleneck that hinders faster rendering is the limited transmission speed of the wireless network we used in the experiment, which slows down drastically when more than 16 workers are added to the compute cluster. We believe that this problem will be alleviated soon as next-generation mobile networks that offer faster data transfer speed are available in the near future. Then, a more sophisticated approach may be required, in which, for example, multiple small-scale mobile clusters alternatively render interleaved frames, effectively increasing the scalability of the presented mobile distributed rendering system. To the best of our knowledge, this work is the first effort to explore a computing cluster made of ubiquitous, easily available mobile devices, for interactively ray tracing large 3D scenes. We believe that the proposed mobile distributed computing scheme can easily be modified or extended for visualizing other kinds of large-scale scientific datasets.

## Figures and Tables

**Table 1:** Memory spaces required by the 3D geometries of example 3D scenes. The sizes of the kd-trees that are produced by the de facto standard algorithm, based on combinations of the tree construction and representation methods [21,22], are compared to those of the actual triangular mesh data, stored in the indexed-face-set format. As expected, the essential kd-tree structure usually introduces significant memory overhead because of the inevitable replication of triangles in the resulting accelerating tree structure.

	No.’s of Triangles	No.’s of Vertices	Size of Mesh (MB)	Size of kd-Tree (MB)
Sponza	279,163	193,372	9.1	13.1
Soda Hall	2,167,474	4,192,793	152.8	86.0
San Miguel	9,963,191	9,278,776	397.2	621.1
Power Plant	12,748,510	10,960,555	480.4	603.7

**Table 2:** Partitioning the scene’s AABB into cubic cells. The scene’s AABB is subdivided into cubic cells whose size is chosen to be as fine as possible, while the number of geometry cells, which intersect with an object (“# of geom. cells”), cannot exceed 65,536 (=$2^{16}$). In the table, “Cell resolution” shows how finely the scene space is tessellated in cubic cells. The found geometry cell is further subdivided into $1024^{3}$ voxels, and "Precision” indicates the voxel size relative to the length of the longest axis of the AABB.

Scene	Cell Resolution	# of Geom. Cells	Precision
Sponza	991×610×415	65,532	$9.85\times 10^{-7}$
Soda Hall	358×317×203	65,319	$2.73\times 10^{-6}$
San Miguel	278×203×318	65,065	$3.07\times 10^{-6}$
Power Plant	616×250×187	65,472	$1.59\times 10^{-6}$

**Table 3:** Size reduction for a triangular mesh in the the indexed-face-set format. The ratio achieved by the presented quantization method is dependent on both the size of mesh and the ratio between the numbers of faces and vertices. See the text for detail.

Scene	Original (MB)	Qunatized (MB)	Compression Ratio
Sponza	9.1	5.4	1.69
Soda Hall	152.8	72.8	2.10
San Miguel	397.2	220.2	1.80
Power Plant	480.4	271.3	1.77

**Table 4:** Image quality comparison (PSNR in dB). The ray-traced images from the quantized meshes are compared to the ground truth images obtained by ray tracing the original meshes, where three combinations of quantizations are examined. The “- no refl/refr” row indicates the PSNR values achieved when the images are produced with the reflection/refraction feature off. All images are rendered at 1024×1024 pixels. See Figure 2 for the adopted camera views.

Scene	POS	POS/NORM	POS/NORM/TEX
Sponza	42.80	42.59	**33.39**
Soda Hall	38.57	**36.29**	N/A
San Miguel	34.94	29.17	**28.69**
- no refl/refr	35.17	35.13	33.50
Power Plant	43.23	**32.63**	N/A
- no refl/refr	43.23	41.10	N/A

**Table 5:** Performances of three kd-tree construction methods. The effectiveness of Seo et al.’s method [7], which is used in the proposed mobile distributed ray tracing system, is compared on the mobile platform to the two previous methods: “standard” [21,22] and “Choi et al.” [29]. (**a**) Size of kd-trees (unit: MB). The figures in parentheses represent the respective compression ratios with respect to the sizes of the “standard” kd-trees. (**b**) Number of kd-tree nodes (unit: thousand). Each triplet of numbers reveals the frequencies of the inner nodes without triangle references, the inner nodes with triangle references and the leaf nodes in the kd-tree. The reduction effect is most evident for the San Miguel scene, leading to the greatest kd-tree compression among the four scenes. (**c**) Ray tracing time (unit: ms). The time for rendering an image of 1024×1024 pixels was measured on a single LG G5 smartphone. As the original triangular meshes were used without quantization in this test, the San Miguel and Power Plant scenes could not be rendered with the standard kd-trees due to the lack of GPU memory space. The percentages in parentheses show how seriously the slight modification of the kd-tree structure affects the ray tracing speed in comparison to Choi et al.’s method.

(**a**)			
**Scene**	**Standard**	**Choi et al.**	**Seo et al.**
Sponza	13.1	8.3 (1.58)	6.6 (1.98)
Soda Hall	86.0	50.1 (1.72)	41.8 (2.06)
San Miguel	621.1	285.3 (2.18)	205.3 (3.03)
Power Plant	603.7	321.0 (1.88)	252.7 (2.39)
(**b**)			
**Scene**		**Choi et al.**	**Seo et al.**
Sponza		211/281/351	203/172/289
Soda Hall		1193/1537/1961	1170/1030/1685
San Miguel		**9696/6392/12,892**	**5183/3014/8197**
Power Plant		7066/10,300/12,216	6934/6553/10,210
(**c**)			
**Scene**	**Standard**	**Choi et al.**	**Seo et al.**
Sponza	2632.4	2711.6	2776.6 (+2.4%)
Soda Hall	2788.1	2801.5	2936.9 (+4.8%)
San Miguel *	-	3489.5	3661.1 (+4.9%)
Power Plant	-	3091.1	3203.0 (+3.6%)

* No texture maps were applied.

**Table 6:** Size reduction effect by the presented scene compression method.

Scene	Original (MB)	Ours (MB)	Compression Ratio
Sponza	22.2	12.0	1.85
Soda Hall	238.8	114.6	2.08
San Miguel	1018.3	425.5	2.39
Power Plant	1084.1	524.0	2.07

**Table 7:** Increases in rendering times due to scene compression. The ray tracing times taken for producing images of 1024×1024 pixels are reported in milliseconds. “T-mesh” and “Kd-tree” indicate the cases when the respective size reduction techniques were applied, and "Both” indicates both techniques were applied.

(**a**) One worker node participated				
	**Original**	**T-Mesh**	**Kd-Tree**	**Both**
Sponza	2632.4	2832.2	2776.6	2905.4
		(+7.6%)	(+5.5%)	(+10.4%)
Soda Hall	2788.1	2893.4	2936.9	3224.5
		(+3.8%)	(+5.3%)	(+15.7%)
San Miguel	–	3487.6	3661.1	4257.8
Power Plant	–	3102.2	3203.0	3862.6
(**b**) 16 worker nodes participated				
	**Original**	**T-Mesh**	**Kd-Tree**	**Both**
Sponza	358.1	366.8	361.9	380.6
		(+2.4%)	(+1.1%)	(+6.3%)
Soda Hall	321.5	343.1	342.3	347.8
		(+6.7%)	(+6.5%)	(+8.2%)
San Miguel	–	409.2	428.5	433.4
Power Plant	–	368.0	378.1	393.8

**Table 8:** Distributed ray tracing times for varying tile sizes. The rendering times for producing a 1024×1024 image using tiles of given pixel sizes are reported in milliseconds. In this experiment, the static load balancing scheme was adopted for distributed ray tracing, which outperformed the dynamic load balancing scheme, as will be explained shortly.

(**a**) One worker node participated				
	**256 × 256**	**128 × 128**	**64 × 64**	**32 × 32**
Sponza	3030.6	2905.4	3903.6	5754.2
Soda Hall	3107.8	3224.5	3839.0	5639.8
San Miguel	6208.7	6128.5	6744.9	8162.8
Power Plant	3939.7	3862.6	4682.2	6845.1
(**b**) 16 worker nodes participated				
	**256×256**	**128 × 128**	**64 × 64**	**32 × 32**
Sponza	374.4	380.6	396.6	522.7
Soda Hall	368.6	347.8	408.2	563.5
San Miguel	678.1	647.5	660.6	698.7
Power Plant	427.4	393.8	459.0	689.8

**Table 9:** Mobile distributed ray tracing times for two load balancing strategies. The times to render an image of 1024×1024 pixels using 128×128 tiles are reported in milliseconds for different numbers of worker nodes that participate in distributed computing. The efficiency numbers in parentheses measure the scalability of our mobile distributed ray tracer.

(**a**) Dynamic load balancing					
	**1**	**2**	**4**	**8**	**16**
Sponza	2763.2	1526.9	892.9	682.4	621.7
	(1.000)	(0.905)	(0.774)	(0.506)	(0.278)
Soda Hall	3184.5	1711.2	937.2	720.8	698.7
	(1.000)	(0.930)	(0.849)	(0.552)	(0.285)
San Miguel	5129.6	2883.7	1658.9	1293.2	1077.3
	(1.000)	(0.889)	(0.761)	(0.496)	(0.298)
Power Plant	3218.5	1799.4	951.3	759.5	724.1
	(1.000)	(0.894)	(0.846)	(0.530)	(0.278)
(**b**) Static load balancing					
	**1**	**2**	**4**	**8**	**16**
Sponza	2905.4	1680.9	896.4	546.5	380.6
	(1.000)	(0.864)	(0.810)	(0.665)	(0.477)
Soda Hall	3224.5	1621.2	876.9	553.1	347.8
	(1.000)	(0.994)	(0.919)	(0.729)	(0.579)
San Miguel	6128.5	3283.8	1828.7	1145.3	647.5
	(1.000)	(0.933)	(0.838)	(0.669)	(0.592)
Power Plant	3862.6	2218.0	1364.9	704.6	393.8
	(1.000)	(0.871)	(0.707)	(0.685)	(0.613)
